# Genome expression analysis by suppression subtractive hybridization identified overexpression of Humanin, a target gene in gastric cancer chemoresistance

**DOI:** 10.1186/2008-2231-22-14

**Published:** 2014-01-08

**Authors:** Negar Mottaghi-Dastjerdi, Mohammad Soltany-Rezaee-Rad, Zargham Sepehrizadeh, Gholamreza Roshandel, Farzaneh Ebrahimifard, Neda Setayesh

**Affiliations:** 1 Department of Pharmaceutical Biotechnology and Pharmaceutical Biotechnology Research Center, School of Pharmacy, Tehran University of Medical Sciences, Tehran 1417614411, Iran; 2 Pharmaceutical Sciences Research Center, Sari School of Pharmacy, Mazandaran University of Medical Sciences, Sari, Iran; 3 Golestan Research Center of Gastroenterology and Hepatology, Golestan University of Medical Sciences, Golestan Iran; 4 Department of General Surgery, School of Medicine, Shahid Beheshti University of Medical Sciences, Tehran Iran

**Keywords:** Apoptosis, Chemoresistance, Gastric cancer, Suppression subtractive hybridization, Humanin

## Abstract

**Background:**

In cancer cells, apoptosis is an important mechanism that influences the outcome of chemotherapy and the development of chemoresistance. To find the genes involved in chemoresistance and the development of gastric cancer, we used the suppression subtractive hybridization method to identify the genes that are overexpressed in gastric cancer tissues compared to normal gastric tissues.

**Results:**

In the suppression subtractive hybridization library we constructed, the most highly overexpressed genes were humanin isoforms. Humanin is a recently identified endogenous peptide that has anti-apoptotic activity and has been selected for further study due to its potential role in the chemoresistance of gastric cancer. Upregulation of humanin isoforms was also observed in clinical samples by using quantitative real-time PCR. Among the studied isoforms, humanin isoform 3, with an expression level of 4.166 ± 1.44 fold, was the most overexpressed isoform in GC.

**Conclusions:**

The overexpression of humanin in gastric cancer suggests a role for chemoresistance and provides new insight into the biology of gastric cancer. We propose that humanin isoforms are novel targets for combating chemoresistance in gastric cancer.

## Background

Gastric cancer (GC) is the fourth most common type of malignancy and the second leading cause of cancer mortality, which has increased in developing countries [1,2]. Different genetic and epigenetic alterations are involved in the development of GC which include alterations in oncogenes (*c-erbB2*), tumor suppressor genes (*p53*), DNA repair genes (*hMLH1*), cell cycle regulators (*cyclin E*), and signaling molecules (*TGFB1/2*) [3,4]. Identification of the molecular mechanisms that contribute to the pathogenesis of GC could help us find targets for early diagnosis, classification, and treatment of it [2]. Although some gene alterations have been identified in GC, the fundamental molecular mechanisms leading to it need to be elucidated [5-7]. Finding the genes that are differentially expressed in GC is one of the best approaches in establishing new biomarkers and therapeutic targets. In addition, these studies could improve our knowledge about molecular biology and carcinogenesis of GC.

Chemotherapy has been an important treatment for gastrointestinal cancers [2], although its success rate is limited due to chemoresistance (e.g. resistance to cisplatin, 5-fluorouracil, mitomycin C or doxorubicin). It is widely accepted that the apoptotic capacity of cancer cells is critical in determining its response to chemotherapeutic agents. The anti-apoptotic nature of cancer cells becomes a mechanism in its chemoresistance, and allows the tumor to survive [8,9]. In addition cell survival processes have an important role in chemoresistance; autophagy has been identified in chemoresistance and is known as a survival factor for tumor cells in the early stages of carcinogenesis. Autophagy is increased by the level of stress but the resulting event varies which could either lead to survival by inhibition of autophagy or to an apoptotic cell death [10]. Many high throughput studies have documented the genes alterations in GC [3,4], although they failed to encompass a complete view of their molecular pathogenesis and chemoresistance. Along with these studies, towards establishing more data about the genes alterations in GC, our research used suppression subtractive hybridization (SSH), a high throughput gene expression analysis method: this requires no prior knowledge about gene selection. The SSH is a method of selective amplification of differentially expressed sequences, which overcomes the technical drawbacks of traditional subtraction methods. Some of the advantages of the SSH method include minute amounts of required initial mRNA, elimination of the need for physical separation of single- and double-stranded molecules, equalization of the abundance of mRNA sequences within the target population and suitable for detection of rare transcripts [11].

In our study, among the identified genes from the constructed SSH library, we found four isoforms of Humanin (HN, HN1 [EMBL: CR612552, GenBank: AC131055, Swiss-Prot: P0CJ68], HN3 [EMBL: AL109955, GenBank: AL135939, Swiss-Prot: P0CJ70], HN6 [EMBL: AC231380, GenBank: AC231380, Swiss-Prot: P0CJ73], and HN10 [EMBL: AL158819, GenBank: AL158819, Swiss-Prot: P0CJ77]) as the overexpressed genes in GC. While it has been demonstrated that Humanin (HN) bears an endogenous synthesis source [12], the precise origin of its gene (or genes) is not specified [13]. HN is a recently identified endogenous peptide that protects cells against cytotoxicity and suppresses apoptosis caused by various stimuli, e.g., serum deprivation, UV irradiation, or staurosporine [13]. The cytoprotective effects of HN seems to be through various mechanisms including its antiapoptotic, metabolic (improvement of the mitochondrial bioactivity) and anti-inflammatory effects [14]. Furthermore, considering the antiapoptotic effects of HN through its binding to Bax, a Bcl-2 family pro-apoptotic protein [15], HN could mask pro-apoptotic effects of chemotherapy agents.

We provide new information about genes associated with the development of GC, particularly those involved in the chemoresistance of cancer cells: this can have a significant influence on treatment for this type of cancer that could be considered as a target in drug discovery in combating chemoresistance in gastric cancer, which typically has a poor prognosis.

## Methods

### Tissue sample preparation

In establishing the main SSH library, both normal and tumor gastric tissues were collected from a 64-year-old male patient during surgery. A pathologist dissected the target tissues under the microscope with special care for minimal contamination of nonepithelial cells, and RNA*later®* (Ambion, Austin, TX, USA) was used to stabilize the RNA during storage. Hematoxylin–eosin (H&E) staining was done on the tissue to determine the tumor type and its degree of invasion.

In order to check the expression of the differentially expressed genes by quantitative real-time PCR (qRT-PCR), ten clinical tissue samples (five tumor and five normal samples) were collected from patients with endoscopy: all samples were obtained prior to chemotherapy. The consent form of The Biologic Sampling Ethics Committee, Tehran University of Medical Sciences (TUMS) was received from each patient before surgery or endoscopy.

### Total RNA extraction

Total RNA was extracted from tissues with the TriPure Isolation Reagent (Roche Applied Science, Indianapolis, IN, USA). Its concentration and purity were analyzed using the Biophotometer (Eppendorf, Hamburg, GY), and its integrity was visually checked with 1% denatured agarose gel.

### mRNA isolation

Isolation of mRNA was done with the DynaBead® mRNA Isolation Kit (Dynal, Lake Success, NY, USA). Briefly, the appropriate amount of DynaBeads oligo (dT)_25_ was equilibrated with 100 μl of binding buffer (100 mM Tris–HCl, 500 mM LiCl, 10 mM EDTA, 1% LiDS, and 5 mM DTT). Diluted total RNA and equilibrated DynaBeads were then mixed and incubated for 5 min at 37°C in a shaking incubator. The beads were washed twice using 200 μl of washing buffer (10 mM Tris–HCl, 0.15 M LiCl, and 1 mM of EDTA). 10 μl of elution buffer was added to the DynaBeads and incubated for 2 min at 67°C. The DynaBeads were placed on the magnet, and the eluted mRNA in supernatant was then isolated. The purified mRNA was checked with 1% denatured agarose gel.

### Suppression subtractive hybridization (SSH)

Using the SSH method, the subtracted library can be created from one sample pair (including cancerous and normal tissues) in both forward and reverse directions, while the expression of the achieved genes are checked in clinical tissue samples with analysis methods that included qRT-PCR [16]. In this study, SSH was carried out with the PCR-Select™ cDNA Subtraction Kit (Clontech, Palo Alto, CA, USA) according to the manufacturer’s protocol. In summary, first- and second-strand cDNA were synthesized using 2 μg mRNA from the gastric cancerous (tester) and normal (driver) tissues, and digested with *Rsa* I. For the reverse subtraction, the tester was used as driver, and the driver was used as tester. Tester cDNA was subdivided into two portions, and special adaptors were added to each. After two hybridizations between the tester and driver (towards eliminating non-altered genes in the two samples), the remaining differentially expressed sequences were amplified with two PCR rounds using *Pwo* enzyme to reduce any background products and to enrich the differentially expressed sequences. For identification of the differentially overexpressed genes, the constructed library (products of the secondary PCR step of SSH) was then cloned and sequenced as the following steps.

### Cloning and confirmation of the positive clones

The secondary PCR product of the SSH method was purified with the PCR Product Purification Kit (Roche Applied Sciences, Indianapolis, IN, USA), cloned into pUC19 plasmid vectors and transformed into *Escherichia coli* NovaBlue competent cells (Novagen, Madison, WI, USA). Randomly selected positive colonies were first confirmed with a colony PCR, using N1 and N2R primers (Table 1). Plasmids from the confirmed positive clones were isolated by the High Pure Plasmid Isolation Kit (Roche Applied Sciences, Indianapolis, IN, USA) and used in single direction DNA sequencing with the BigDye Terminator Version 3.1 Sequencing Kit and a 3730xl Automated Sequencer (Applied Biosystems, Foster City, CA, USA). To identify these sequences, similarity searches were carried out with BLAST ( http://blast.ncbi.nlm.nih.gov/Blast.cgi).

**Table 1:** Designed primers sequences used to quantify gene expression by real-time PCR

**Primer name**	**Accession number**	**Length**	**Sequence (5′ to 3′)**	**Annealing temp**	**Location**	**Product size**
HN1-F	NM_001190452.1	20	CGCAGGCCCTAAACTACCAG	61°C	1091-1110	206 bp
HN1-R	NM_001190452.1	20	TGCTACTGTCGATGTGGACC		1277-1296	
HN3-F	NM_001190472.1	20	GGTGATAGCTGGCTGGCTTA	59°C	180-199	164 bp
HN3-R	NM_001190472.1	20	ATTAGTGGCTGCTTTTGGGC		324-343	
HN6-F	NM_001190487.1	20	TTTACCCAGGCGCAGTGGAC	62°C	60-79	247 bp
HN6-R	NM_001190487.1	22	GGCTCAGTAGGCTTATCACCAC		285-306	
HN10-F	NM_001190708.1	23	CGAGAAGACCCTATGTATGGAGC	61°C	603-625	137 bp
HN10-R	NM_001190708.1	20	AGGTTGCTCGGAGGTTGAAT		720-739	
N1	Based Ligated Adaptor	22	TCGAGCGGCCGCCCGGGCAGGT	68°C		
N2-R	Based Ligated Adaptor	20	AGCGTGGTCGCGGCCGAGGT			
ACTB-F	NM_001101.3	21	ATGGCCACGGCTGCTTCCAGC	60°C	763-783	322 bp
ACTB-R	NM_001101.3	21	CAGGAGGAGCAATGATCTTGA		1064-1084	

### Analysis of the subtraction efficiency

Real-time PCR was used to estimate the efficiency of subtraction by comparing the abundance of a non-differentially expressed gene (a housekeeping gene: beta actin) before and after subtraction. Reactions were prepared by adding 10 μl of SYBR Premix Ex Taq (Takara, Kusatsu, Japan), 1 μl of the sample, 0.8 μl of the primers (10 μM), 0.4 μl of ROX dye and DEPC-treated water to a final volume of 20 μl. The thermal program for the reaction cycles was 10 min at 95°C, followed by 40 cycles of 30 sec at 95°C, and 1 min at 60°C. Melting curve analysis was done by increasing the temperature from 65°C to 95°C in 0.1°C/sec increments for each fluorescence acquisition, using the Step-One-Plus Apparatus (Applied Biosystems, Foster City, CA, USA). Relative expression of the beta actin gene in the subtracted and non-subtracted samples was used in the calculation of subtraction efficiency.

### Quantitative real-time PCR (qRT-PCR)

Quantitative expression analysis was performed with real-time PCR (Applied Biosystems, Foster City, CA, USA) for the SSH-identified HN isoforms. Primer design was done with PrimerSelect, Version 7.1.0 (DNAstar, Madison, WI, USA) and synthesized by the TAG Company (TAG Copenhagen, Copenhagen, Denmark) (Table 1). The qRT-PCR was run with SYBR Premix Ex Taq (Takara, Kusatsu, Japan) in a final volume of 20 μl, containing 2X master mix 10 μl, each with forward and reverse primers (10 μM) 1 μl, ROX dye II 0.4 μl, and cDNA 2 μl.

The PCR thermocycle program was set at 95°C for 10 min followed by 40 cycles of 95°C for 30 sec, 62°C for 30 sec. Melt curve analysis followed the PCR step and increased the temperature from 65°C to 95°C, with 0.1°C/sec increments in each fluorescence reading.

### Statistical analysis

The relative gene expression of HN isoforms in tumor and normal tissues was analyzed using the Livak method [17]. The statistical significance was set at P < 0.05.

## Results

### Histological examination

**Figure 1:**
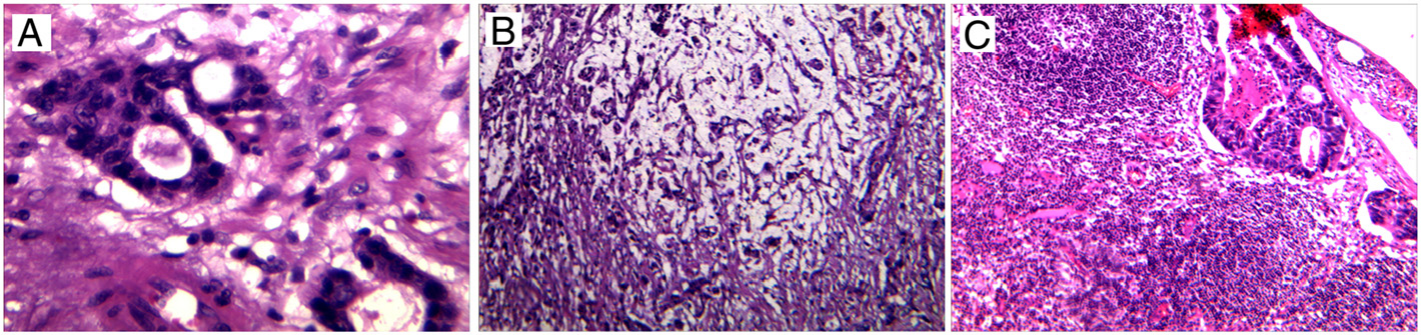
**Histological features of the resected gastric cancerous tissue stained by H&E to determine the cancer type and metastasis. (A)** Tubular structure formation, which characterizes these cancerous cells as an adenocarcinoma tumor type. **(B)** Mucin producing type of GC could be determined by the white matrix surrounded the cancerous cells. **(C)** Cross-sectional analysis of a pre-gastric lymph node. The tubular formation of the gastric adenocarcinoma cells and lymph node follicles could be observed.

Histological results indicated that the tumor was a moderately differentiated, mucin-producing type of gastric adenocarcinoma, located in the prepyloric area. Local invasion to the lymph node was observed in two of the six pre-gastric lymph nodes (Figure 1).

### Suppression subtractive hybridization (SSH)

**Figure 2:**
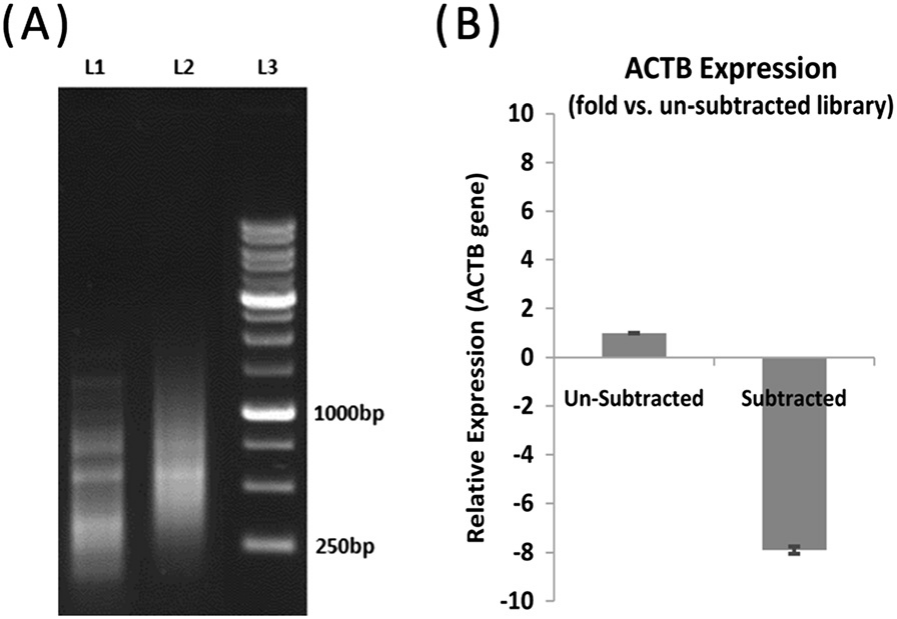
**Constructed suppression subtractive hybridization library. (A)** Forward library reveals differentially expressed genes. Lane 1: Primary PCR enrichment, Lane 2: Secondary PCR enrichment, Lane 3: Ladder. **(B)** Analysis of the subtraction efficiency. The relative expression levels of the beta actin gene in the non-subtracted and subtracted samples indicate that there is an 8.9-fold decrease in beta actin expression in the subtracted cDNAs.

Cloning of the two subtracted libraries (forward and reverse), ranged from 100–800 base pairs in size (Figure 2A), resulted in 120 clones. Among the overexpressed genes from the forward library, three clones had sequences, which were identical to four isoforms of HN; HN1, HN3, HN6, and HN10. They were selected for qRT-PCR analysis due to their probable role in the chemoresistance of GC cells.

### Subtraction efficiency

Real-time PCR analysis demonstrated that beta actin has an 8.9-fold reduction in the subtracted library, compared with the non-subtracted library (Figure 2B). This resulting reduction verified the accuracy of the applied method in finding differentially expressed genes in GC.

### Quantitative real-time PCR (qRT-PCR)

**Figure 3:**
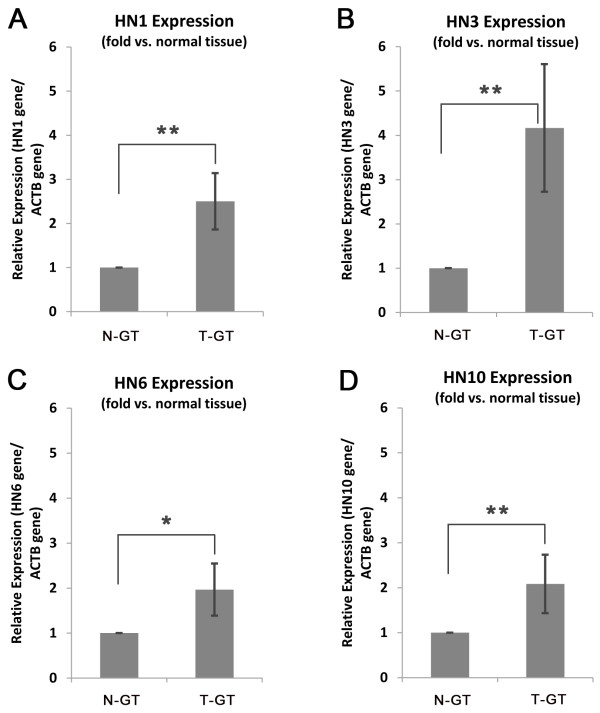
**Relative gene expression analysis of HN isoforms in normal and tumor tissue samples, using qRT-PCR. (A)** HN1 isoform. **(B)** HN3 isoform. **(C)** HN6 isoform. **(D)** HN10 isoform. N-GT: normal gastric tissue, T-GT: tumor gastric tissue. Whiskers represent the STDEV of expression in samples (n = 10). *P < 0.05, **P < 0.01.

To confirm the results obtained from the constructed SSH library, relative gene expression of HN isoforms was checked in some endoscopic tissue samples. The results showed overexpression of HN isoforms in clinical tissue samples (Figure 3). These results confirmed the efficiency of the SSH library, which also indicated that these isoforms were significantly overexpressed in GC. Between the studied isoforms, HN3 with an expression level of 4.166 ± 1.44 fold was the most overexpressed isoform in GC.

## Discussion

This study focused on the overexpressed genes associated with gastric adenocarcinoma as the most prevalent and life-threatening type of cancer in Iran [18]. Chemoresistance of tumor cells is a therapeutic defeat that affects treatment outcomes with cancer [19]. Development of resistance is a common occurrence in GC, with apoptosis considered to be one of the major mechanisms in tumorigenesis and chemoresistance of cells [9,19]. Two important mechanisms involved in this resistance are the loss of pro-apoptotic signals and the gain of anti-apoptotic mechanisms [20].

Using SSH in this study, HN isoforms, the anti-apoptotic endogenous peptides with a potential role in the chemoresistance of GC cells were identified. Furthermore, upregulation of identified HN isoforms were confirmed using qRT-PCR. The importance of this study is the specific isoforms of HN identified as the overexpressed genes in GC (HN isoforms 1, 3, 6 and 10). Due to the high similarities between HN isoforms and lack of isoform-specific antibodies, detection of HN isoforms at the protein level by western blotting or IHC was not provided in this study.

Our study identified the HN gene as an overexpressed gene in GC. It is a newly discovered 24-amino acid peptide; with 75 bases in an open reading frame and 950 bases downstream of the 5′ end of the HN cDNA (HN cDNA is 99% similar to mitochondrial DNA) [21,22]. Recently, studies have shown that HN is specifically bound to BAX, tBID, and BimEL and executes its anti-apoptotic activity through selective attachment to BAX and translocation inhibition of BAX to the mitochondria [15,23,24]. There have been many *in vitro* studies demonstrating the protective characteristic of HN in different cell types [25-29]. The results suggest that HN could increase the energy produced by mitochondria [30]. Furthermore, HN could similarly increase the ATP- vs. pyrovate-biogenesis, which leads to the assumption that HN may have an important role in mitochondrial dysfunction-related diseases, including cancer [23,30,31]. HN overexpression in GC could be related to stress in a microenvironment of cancer cells (e.g., nutrient deprivation) that triggers apoptosis [24]. Cancer cells, along with the upregulation of the HN gene as an anti-apoptotic factor, combated apoptosis in them. In addition, nutrient deprivation and continuous cell division demanded additional energy resources. In this way, HN as a function of ATP production in cancer cells could diminish metabolic stresses.

Our results showed that HN3, with a 4.166 ± 1.44 -fold increase in GC tissues, is the dominant isoform. HN isoforms have a unique coding sequence for the HN peptide. Various isoforms in the HN gene with different 5′-UTR and 3′-UTR might have probable roles in the stability of its peptide. Peptides with the highest stability, with increased residence time in cancer cells, could also have more influence in tumorigenesis and chemoresistance [32].

Our results suggest that upregulation of HN in GC could be an important molecular event in its tumorigenesis. Given its anti-apoptotic activity in cancer cells, it could be one of the fundamental mechanisms in chemoresistance of GC cells: upregulation of HN alleviates metabolic stresses by ATP production which could have an important role in the early stages of tumorigenesis. HN can potentially serve as a new biomarker in the diagnosis of GC since it is present in blood circulation. To date, HN was not considered a key gene in the chemoresistance of tumor cells; future studies that target HN in gastric chemoresistance cells may have a valuable impact on the therapeutic modality used for this cancer.

## Conclusions

In conclusion, using the SSH method, the overexpression of HN 1, 3, 6, and 10 isoforms were identified for the first time in gastric cancer cells. Considering the fundamental role of anti-apoptosis in the chemoresistance of cancer cells and the high expression level of HN in GC, further studies are needed to evaluate the role of HN isoforms and chemoresistance. In addition, since overexpression of HN isoforms could lead to chemoresistance in GC this gene could be a candidate in drug discovery investigations for targeting chemoresistance in this cancer.

## Competing interests

All authors declare that they have no competing interests.

## Authors’ contributions

NMD carried out the construction of suppression subtractive hybridization and helped to draft the manuscript. MSRR carried out the qRT-PCR analysis. NS participated in study design and helped to draft the manuscript. GR participated in collecting endoscopic normal and tumor tissues. FE participated in resection of SSH pair samples (tumor and normal tissues) by surgery. ZS conceived of the study, and participated in its design and coordination and helped to draft the manuscript. All authors read and approved the final manuscript.

## References

[B1] Bray F Ren JS Masuyer E Ferlay J Global estimates of cancer prevalence for 27 sites in the adult population in 2008 Int J Cancer 2013 132 5 1133 1145 10.1002/ijc.27711 22752881

[B2] Layke JC Lopez PP Gastric cancer: diagnosis and treatment options Am Fam Physician 2004 69 5 1133 15023013

[B3] Yamashita K Sakuramoto S Watanabe M Genomic and epigenetic profiles of gastric cancer: potential diagnostic and therapeutic applications Surg Today 2011 41 1 24 38 10.1007/s00595-010-4370-5 21191688

[B4] Nagini S Carcinoma of the stomach: a review of epidemiology, pathogenesis, molecular genetics and chemoprevention World J Gastrointest Oncol 2012 4 7 156 10.4251/wjgo.v4.i7.156 22844547 PMC3406280

[B5] Hussein NR Helicobacter pylori and gastric cancer in the Middle East: a new enigma? WJG 2010 16 26 3226 10.3748/wjg.v16.i26.3226 20614477 PMC2900713

[B6] Wu CW Chen GD Fann CSJ Lee AFY Chi CW Liu JM Weier U Chen JY Clinical implications of chromosomal abnormalities in gastric adenocarcinomas Genes Chromosom Cancer 2002 35 3 219 231 10.1002/gcc.10106 12353264

[B7] Hou Q Wu YH Grabsch H Zhu Y Leong SH Ganesan K Cross D Tan LK Tao J Gopalakrishnan V Integrative genomics identifies RAB23 as an invasion mediator gene in diffuse-type gastric cancer Cancer Res 2008 68 12 4623 4630 10.1158/0008-5472.CAN-07-5870 18559507

[B8] Wilson T Longley D Johnston P Chemoresistance in solid tumours Ann Oncol 2006 17 suppl 10 315 324 10.1093/annonc/mdl280 17018746

[B9] Oki E Baba H Tokunaga E Nakamura T Ueda N Futatsugi M Mashino K Yamamoto M Ikebe M Kakeji Y Maehara Y Akt phosphorylation associates with LOH of PTEN and leads to chemoresistance for gastric cancer Int J Cancer 2005 117 3 376 380 10.1002/ijc.21170 15900596

[B10] Tyson JJ Baumann WT Chen C Verdugo A Tavassoly I Wang Y Weiner LM Clarke R Dynamic modelling of oestrogen signalling and cell fate in breast cancer cells Nat Rev Cancer 2011 11 7 523 532 10.1038/nrc3081 21677677 PMC3294292

[B11] Diatchenko L Lau Y Campbell AP Chenchik A Moqadam F Huang B Lukyanov S Lukyanov K Gurskaya N Sverdlov ED Suppression subtractive hybridization: a method for generating differentially regulated or tissue-specific cDNA probes and libraries Proc Natl Acad Sci 1996 93 12 6025 6030 10.1073/pnas.93.12.6025 8650213 PMC39182

[B12] Tajima H Niikura T Hashimoto Y Ito Y Kita Y Terashita K Yamazaki K Koto A Aiso S Nishimoto I Evidence for in vivo production of Humanin peptide, a neuroprotective factor against Alzheimer’s disease-related insults Neurosci Lett 2002 324 3 227 231 10.1016/S0304-3940(02)00199-4 12009529

[B13] Bodzioch M Lapicka-Bodzioch K Zapala B Kamysz W Kiec-Wilk B Dembinska-Kiec A Evidence for potential functionality of nuclearly-encoded humanin isoforms Genomics 2009 94 4 247 256 10.1016/j.ygeno.2009.05.006 19477263

[B14] Zapala B Kaczynski L Kiec-Wilk B Staszel T Knapp A Thoresen GH Wybranska I Dembinska-Kiec A Humanins, the neuroprotective and cytoprotective peptides with antiapoptotic and anti-inflammatory properties Pharmacol Rep 2010 62 5 767 777 21098860 10.1016/s1734-1140(10)70337-6

[B15] Guo B Zhai D Cabezas E Welsh K Nouraini S Satterthwait AC Reed JC Humanin peptide suppresses apoptosis by interfering with Bax activation Nature 2003 423 6938 456 461 10.1038/nature01627 12732850

[B16] Diatchenko L Lukyanov S Lau Y-FC Siebert PD [20] Suppression subtractive hybridization: a versatile method for identifying differentially expressed genes Methods Enzymol 1999 303 349 380 10349654 10.1016/s0076-6879(99)03022-0

[B17] Livak KJ Schmittgen TD Analysis of relative gene expression data using real-time quantitative PCR and the $2^{-\Delta \Delta C_{T}}$ Method Methods 2001 25 4 402 408 10.1006/meth.2001.1262 11846609

[B18] Malekzadeh R Derakhshan MH Malekzadeh Z Gastric cancer in Iran: epidemiology and risk factors Arch Iran Med 2009 12 6 576 583 19877751

[B19] Hajra KM Tan L Liu JR Coukos G, Berchuck A, Ozols R Defective apoptosis underlies chemoresistance in ovarian cancer Ovarian Cancer. Volume 622 2008 New York: Springer 197 208 10.1007/978-0-387-68969-2_16 18546629

[B20] Reuter S Eifes S Dicato M Aggarwal BB Diederich M Modulation of anti-apoptotic and survival pathways by curcumin as a strategy to induce apoptosis in cancer cells Biochem Pharmacol 2008 76 11 1340 1351 10.1016/j.bcp.2008.07.031 18755156

[B21] Lee C Yen K Cohen P Humanin: a harbinger of mitochondrial-derived peptides? Trends Endocrinol Metab 2013 10.1016/j.tem.2013.01.005 PMC3641182 23402768

[B22] Nishimoto I Matsuoka M Niikura T Unravelling the role of Humanin Trends Mol Med 2004 10 3 102 105 10.1016/j.molmed.2004.01.001 15106598

[B23] Yen K Lee C Mehta H Cohen P The emerging role of the mitochondrial-derived peptide humanin in stress resistance J Mol Endocrinol 2013 50 1 R11 R19 10.1530/JME-12-0203 23239898 PMC3705736

[B24] Kariya S Takahashi N Ooba N Kawahara M Nakayama H Ueno S Humanin inhibits cell death of serum-deprived PC12h cells Neuroreport 2002 13 6 903 907 10.1097/00001756-200205070-00034 11997711

[B25] Hashimoto Y Ito Y Niikura T Shao Z Hata M Oyama F Nishimoto I Mechanisms of neuroprotection by a novel rescue factor humanin from Swedish mutant amyloid precursor protein Biochem Biophys Res Commun 2001 283 2 460 468 10.1006/bbrc.2001.4765 11327724

[B26] Hashimoto Y Niikura T Chiba T Tsukamoto E Kadowaki H Nishitoh H Yamagishi Y Ishizaka M Yamada M Nawa M The cytoplasmic domain of Alzheimer’s amyloid-β protein precursor causes sustained apoptosis signal-regulating kinase 1/c-Jun NH2-terminal kinase-mediated neurotoxic signal via dimerization J Pharmacol Exp Ther 2003 306 3 889 902 10.1124/jpet.103.051383 12829723

[B27] Ikonen M Liu B Hashimoto Y Ma L Lee K-W Niikura T Nishimoto I Cohen P Interaction between the Alzheimer’s survival peptide humanin and insulin-like growth factor-binding protein 3 regulates cell survival and apoptosis Proc Natl Acad Sci 2003 100 22 13042 13047 10.1073/pnas.2135111100 14561895 PMC240741

[B28] Jung SS Van Nostrand WE Humanin rescues human cerebrovascular smooth muscle cells from Aβ‐induced toxicity J Neurochem 2003 84 2 266 272 10.1046/j.1471-4159.2003.01524.x 12558989

[B29] Wang D Li H Yuan H Zheng M Bai C Chen L Humanin delays apoptosis in K562 cells by downregulation of P38 MAP kinase Apoptosis 2005 10 5 963 971 10.1007/s10495-005-1191-x 16151632

[B30] Kariya S Takahashi N Hirano M Ueno S Humanin improves impaired metabolic activity and prolongs survival of serum-deprived human lymphocytes Mol Cell Biochem 2003 254 1–2 83 89 14674685 10.1023/a:1027372519726

[B31] Kariya S Hirano M Furiya Y Ueno S Effect of humanin on decreased ATP levels of human lymphocytes harboring A3243G mutant mitochondrial DNA Neuropeptides 2005 39 2 97 101 10.1016/j.npep.2004.11.004 15752543

[B32] Audic Y Hartley RS Post‐transcriptional regulation in cancer Biol Cell 2004 96 7 479 498 10.1016/j.biolcel.2004.05.002 15380615

